# Using weight-for-age for predicting wasted children in Cameroon

**DOI:** 10.11604/pamj.2013.14.96.1914

**Published:** 2013-03-11

**Authors:** Georges Nguefack-Tsague, Agatha Tanya Nguti Kien, Charles Ntungwen Fokunang

**Affiliations:** 1 Department of Public Health, Faculty of Medicine and Biomedical Sciences, University of Yaoundé 1, Cameroon; 2 Department of Pharmaceutical Sciences and Traditional Pharmacopoiea, Faculty of Medicine and Biomedical Sciences, University of Yaoundé 1, Cameroon

**Keywords:** Anthropometric measures, nutritional status, discriminant analysis, underweight, wasting

## Abstract

**Introduction:**

The equipment for taking body weights (scales) are more frequently used in Cameroon health centres than measuring boards for heights. Even when the later exist there are some difficulties inherent in their qualities; thus the height measurement is not always available or accurate. Our objective for this study was to construct statistical models for predicting wasting from weight-for-age.

**Methods:**

3742 children aged 0 to 59 months were enrolled in a cross-sectional household survey (2004 Cameroon Demographic and Health Surveys (DHS)) covering the entire Cameroon national territory.

**Results:**

There were highly significant association between underweight and wasting. For all discriminant statistical methods used, the test error rates (using an independent testing sample) were less than 5%; the Area Under Curve (AUC) using the Receiver Operating Characteristic (ROC) was 0.86.

**Conclusion:**

The study showed that weight-for-age can be used for accurately classifying a child whose wasting status is unknown. The result is useful in Cameroon as too often the height measurements may not be feasible, thus the need for estimating wasted children. This study provides baseline information that will help to design a preliminary pivotal study on an immediate nutrition intervention for acute undernutrition. Its complications that could lead to morbidity and mortality can be greatly reduced or set up a management control strategy that will go a long way in reducing the cost of health care in Cameroon.

## Introduction

Childhood undernutrition is one of the important health problems in developing countries. More than 3.6 million mothers and children die each year as a result of undernutrition [1]. The evaluation of young children's nutritional status uses the Z-score which measures the distance, expressed in standard deviations of the standard (reference) population between the individual's anthropometry and the median of the standard (reference) population. The most frequent anthropometric indicators are height-for-age, weight-for-height and weight-for-age. A Z-score greater than 2 would indicate over-nourishment with respect to the corresponding anthropometric measurements. Deficits on these indicators (measured by their values less than -2 standard deviations below the median based on the 2006 WHO Child Growth Standards [2], that is both severe and moderate status for each of the above anthropometric measurements) are known as stunting, wasting and underweight respectively. Some criticisms have been raised regarding the reliability of these indicators. These include the fact that the assumption that the expected weight for a given height does not depend on age is unjustified for constructing the weight-for-height index [3–5]. For instance, Cole [3–5] proposed an age-standardized version of weight-for-height. For the 2006 WHO Child Growth Standards, the standard population is an international sample which is constituted of Brazil, Ghana, India, Norway, Oman and the United States of America (USA), whereas the reference one for NCHS/CDC/WHO is constituted solely of the USA sample population [6–8].

The Millennium Development Goals (MDG) focus on fighting against poverty, mortality, morbidity and undernutrition [9]. One of the aims is to reduce by half the prevalence of underweight among children by 2015. Such an initiative requires the identification of the determinants of undernutrition for the design of appropriate policy interventions.

We were not interested here in the determinants of undernutrition; rather on relationships between the anthropometric measurements. From a practical point of view, apart from the data on age that are easily obtained from recorded files, equipments are required to perform anthropometric assessment. For weight and height, the most common types of equipment used are scales and measurement boards. In Cameroon, electronic scales are available in most health centres. However, the measurement boards for height are not always available, making it difficult to have data on height. Baby balances that are calibrated for length or height are not always readily available in these health centres. Given that high association has been reported between underweight and wasting [6, 7, 17, 18, 24], the main objective of this study was to find out statistical discriminant models to predict wasted children from their weight-for-age scores in the absence of height measurements.

## Methods

Data for 3742 children aged 0 to 59 months were obtained from the 2004 Cameroon Demographic and Health Survey (DHS) that was carried out by Cameroon's National Institute of Statistics and ORC Macro (USA). The sample of Cameroon's DHS was a nationally representative household survey that used a two-stage stratified random sampling design. The first stage was the selection of primary sampling units (PSU) within each of the 22 strata and the second was the selection of households within each PSU. The survey consisted of a household questionnaire and a women's questionnaire. On the average, 22 households were selected in an urban PSU and 28 in a rural PSU for a total of 11584 households. Women aged 15-49 years identified in the households were interviewed. The women's questionnaire contained information on several topics including: background characteristics, contraceptive knowledge and use, maternity and breastfeeding, immunization of children, diarrhea, fever, and cough in children, marriage, fertility preferences, husband's background, work status, age, height and weight of children. A more general description of the DHS methodology can be found in Rutstein and Rojas [18].

The methodology for computing anthropometric indicators was based on the 2006 WHO Child Growth Standards. Statistical models used include the following discriminant analysis: Linear Discriminant Analysis (LDA), Quadratic Discriminant Analysis (QDA), K-Nearest Neighbour (KNN), Epanechnikov Kernel Discriminant (EKD), Normal Kernel Discriminant (NKD), Logistic Discriminant Analysis (LDA), Support Vector Machine (SVM), Neural networks (NNET), Mixture Discriminant Analysis (MDA), Flexible Discriminant Analysis (FDA), Multivariate Adaptive Regression Splines (MARS) and Adaptive Backfitting (BRUTO). The K-nearest neighbour (K-NN) discriminant uses K = 3. The method is relatively insensitive to the choice of K, especially for large samples [19]. This was the case in our application; different values of K did not lead to substantial differences. Kernel nonparametric discriminant method such as Epanechnikov and normal were used with radius 0.3. Varying the radius did not substantially modify the result. Other kernel methods such as uniform, biweight and triweight yield similar results. Logistic regression was used to model the probability of being wasted. A description of these discriminant methods can be found in Hastie et al. [20].

Discriminant Analysis (DA) is a multivariate technique designed to find boundaries that separate items belonging to two or more known groups [19]. It provides a different perspective of viewing wasting as a one-dimensional summary of weight-for-age (underweight). The coding was: wasting=underweight=1; not-wasting=not-underweight=0. The response variable was wasting status whereas the predictor was weight-for-age. The sensibility measures the ability of the technique to correctly classify wasted children. It is the percentage of wasted children that the technique correctly classifies as such. The specificity measures the ability of the technique to correctly classify not wasted children. It is the percentage of not wasted children that the technique correctly classifies as not wasted children. True positive (TP) is the number of wasted children correctly classified; False negative (FN) is the number of wasted children incorrectly classified; True negative (TN) is the number of not wasted children correctly classified and False positive (FP) is the number of not wasted children incorrectly classified.

Ideally, one would like the sensibility and specificity to be 1. However it is not uncommon to have higher sensitivity but lower specificity (or the reverse). A receiver-operator characteristic (ROC) curve helps to visualize and understand the magnitude of the tradeoff between sensitivity and specificity, especially when the technique involves making a choice between continuum cut-off values. Because of the tradeoff between sensitivity and specificity, the Area Under the Curve (AUC) and the error rate are common tools for measuring the quality a discriminant model. A perfect model has AUC of 1 and no error rate.

In order to determining the test error rate, we randomly split the data (3742 children) in two, two thirds (2495) for estimating the discriminant model and one third (1247) for prediction. This avoided using the same data for estimation and prediction.

Statistical analyses were performed with the Statistical Analysis Systems statistical software package version 6.12 (SAS Institute, Cary, NC, USA) [21] and R (version 2.14.1) [22].

## Results

**Figure 1:**
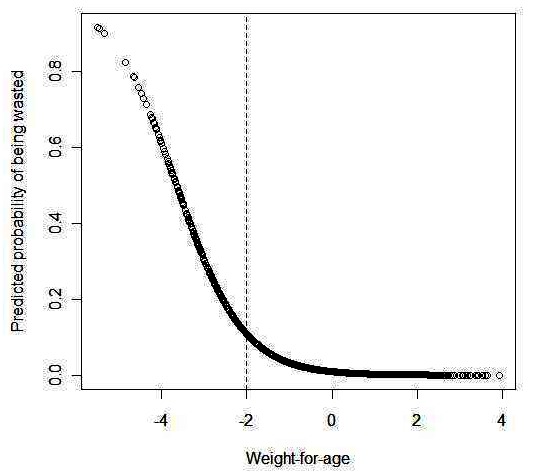
Predicted probability of being wasted using logistic regression as function of weight-for-age

Wasting was observed in 5.5% of children and underweight in 12.9% (Figure 1). As expected, Figure 1 shows that the predicted probability of being wasted (using logistic regression) decreased as the height-for-age score increased. Pearson's correlation between weight-for-age and weight-for-height was 0.6. Underweight status was associated with wasting (Pearson's chi-squared = 534.4, P=0) (Table 1)

**Table 1:** Discriminant analysis methods for wasting as function of weight-for-age

Discriminant Method	TP*	FP	FN	TN	Se	Sp	Test Error rate
Linear Discriminant Analysis (LDA)	13	8	45	1181	0.224	0.993	0.043
Quadratic Discriminant Analysis (QDA)	13	9	45	1180	0.224	0.992	0.043
K-Nearest Neighbour (KNN)	17	16	41	1173	0.293	0.987	0.046
Epanechnikov Kernel Discriminant (EKD)	13	8	45	1181	0.224	0.993	0.043
Normal Kernel Discriminant (NKD)	9	2	49	1187	0.155	0.998	0.041
Logistic Discriminant Analysis (LDA)	13	9	45	1180	0.224	0.992	0.043
Support Vector Machine (SVM)	9	3	49	1186	0.155	0.997	0.042
Neural networks (NNET)	11	8	47	1181	0.190	0.993	0.040
Mixture Discriminant Analysis (MDA)	15	13	46	1176	0.259	0.989	0.045
Flexible Discriminant Analysis (FDA)	13	8	45	1181	0.224	0.993	0.043
Multivariate Adaptive Regression Splines (MARS)	14	12	44	1177	0.241	0.990	0.045
Adaptive Backfitting (BRUTO)	13	9	45	1180	0.224	0.992	0.043

* True Positive (TP), False Positive (FP), False Negative (FN), True Negative (TN), Sensitivity (Se), Specificity (Sp)

**Figure 2:**
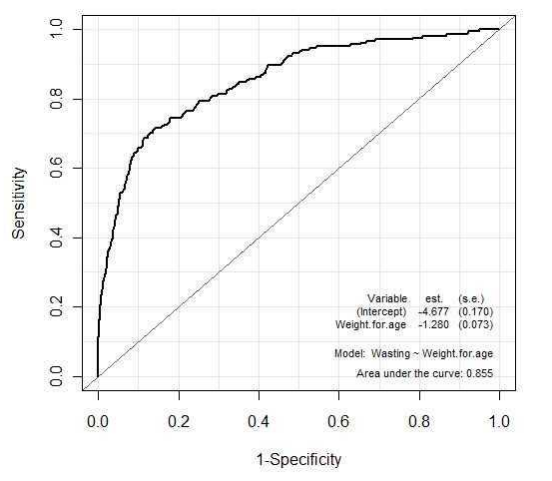
Receiver Operating Caracteristics (ROC) Curve for Wasting Status

The comparison between true wasting status (from data) and the predicted wasting status (using DA) showed that the agreement was excellent between both as evidenced by low error rates in Table 1. The grouping of children given in the data was almost identical to that obtained using DA, using an independent sample of children. The predictive ability of various discriminant analysis methods was demonstrated by their test error rate less than 5%. Figure 2 showed an AUC of 0.86, providing evidence of a good classification.

## Discussion

According to WHO classification, the 5.5% observed for wasting and 12.9% for underweight were both considered medium. These figures were slightly different from the 5.0% and 13.2% respectively for wasting and underweight that were obtained using the NCHS/CDC/WHO international reference standard worldwide used prior to year 2006.

The results of these studies only apply to this sample and associations may change depending on sample′s settings, age range, and prevalence rates. Thus a multi-country study could be carried out to find out how the results would vary across countries. One could also perform an ecological study across selected countries (or all countries with similar available data). However, the drawbacks of such studies are well known. The results may be very different at the individual level. It is well known that ecological correlation studies should be interpreted with caution [23]. De Onís et al. [24] performed an ecological correlation analysis of 22 African countries and found that the prevalence of underweight was positively associated with wasting and stunting and there was a very low correlation between wasting and stunting. Victora [25] showed similar results for others African countries. Similar results could also be found in Centers for Disease Control and Prevention [26]. Blössner et al. [27] in an ecological study using 122 countries also developed a simple regression model using least squares method to predict the prevalence of stunting from the prevalence of underweight. Their models performed well only for the region of Asia and the prediction was not reliable for other regions. In related studies with thirty-eight anthropometric surveys of Brazilian children aged up to 5 years, Victora et al.[28] used the prevalence of weight deficits to estimate that of height deficits with a linear model and concluded that anthropometric surveys as conducted in Brazil, in the context of health services, can be simplified by measuring weight only, instead of both weight and height.

Various factors can affect undernutrition: inadequate intake of vitamins and minerals, suboptimal breastfeeding and complementary feeding practices, socioeconomic factors, demographic and environmental characteristics, food intake that is continuously insufficient to meet dietary energy requirements, poor absorption and/or poor biological use of nutrients consumed may affect undernutrition. There is a sizeable literature on the determinants of undernutrition using anthropometric indicators as dependent variables [10–16]. It is important to note that two other (less popular) methods for evaluating the children's nutritional status are the percentage of the median and the percentile methods. WHO Child Growth Standards can serve as a diagnostic tool to assess unhealthy trends. In addition, the standard could help in monitoring the nutritional status of communities and alert practitioners and policymakers to unhealthy trends in the population. Stunting status, usually referred as chronic (past) undernutrition (growth retardation) represents a measure of long term effects of malnutrition in a population and does not vary significantly according to the season of data collection [17]. Wasting status, usually referred to as acute (current) undernutrition (emaciation) is appropriate for examining short-term effects such as seasonal changes in food supply or short-term nutritional status due to a factor like an illness. Underweight takes into account both chronic and acute undernutrition and is usually used to monitor nutritional status on a longitudinal basis. This suggests that the choice of a particular anthropometric indicator depends on the goal of the policy maker; that is no single index is to be used for all situations.

## Conclusion

In Cameroon, measuring boards for height measurements of children are not always available in some health centres. The weight-for-age scores can be useful for predicting the wasting status of children in these centres. This study provides baseline information that will help to design a preliminary pivotal study on an immediate nutrition intervention for acute undernutrition. Its complications that could lead to morbidity and mortality can be greatly reduced or set up a management control strategy that will go a long way in reducing the cost of health care in Cameroon.
